# Unveiling the Role of the Cellular Tumor Microenvironment and the Therapeutic Targets it Provides in Cutaneous T-Cell Lymphoma

**DOI:** 10.1007/s11912-025-01646-6

**Published:** 2025-03-08

**Authors:** Nikolaos A. Chinas, Stella Kaliampou, Vasiliki Nikolaou

**Affiliations:** 1st Department of Dermatology-Venereology, Medical School, National and Kapodistrian University of Athens, “Andreas Sygros” Hospital for Skin & Venereal Diseases, Athens, Greece

**Keywords:** CTCL, Mycosis Fungoides, Sézary Syndrome, Tumor Microenvironment, Cells, Therapeutic Targets

## Abstract

**Purpose of Review:**

Cutaneous T-Cell Lymphoma (CTCL) poses challenges both in diagnosis and prognosis. The purpose of this review is to address the role of profiling immune and non-immune cells in the tumor microenvironment (TME) as it provides information for better diagnosis, prognosis, biomarker discovery, and personalized treatment strategies.

**Recent Findings:**

Recent evidence suggests that the progression of CTCL is closely linked to the Tumor Microenvironment (TME) which comprises various cell types including immune cells, stromal cells, blood vessels, and the extracellular matrix. Cell profiling within the TME demonstrates the perplexity of intracellular communication of the different cell fates and their mediators as the disease progresses.

**Summary:**

CTCL as a rare form of non-Hodgkin lymphoma often misdiagnosed due to its similarity to other skin conditions. It encompasses diseases like Mycosis fungoides (MF) and Sézary Syndrome (SS), with the latter being more severe. Advances in studying the TME have shown its pivotal role in CTCL progression, highlighting the need for comprehensive cell profiling to enhance diagnosis, prognosis, and treatment personalization.

## Introduction

Cutaneous T-cell lymphomas (CTCLs) encompass a spectrum of non-Hodgkin lymphomas primarily affecting the skin without evidence of extracutaneous involvement at the time of diagnosis. Mycosis fungoides (MF) and its leukemic variant Sezary Syndrome are the most prevalent subtypes [1]. Due to their clinical similarity with inflammatory skin diseases, like psoriasis and eczema, the diagnosis is often challenging. Clinicopathological correlation is always key to the diagnosis with skin biopsies of CTCL be characterized by the infiltration of atypical CD4 + T-cells (CD45RA-, CDw29+) [2, 3]. Recent advancements in single-cell RNA sequencing (sc-RNA seq) have revolutionized our ability to identify malignant T cell clones, thereby enhancing diagnostic accuracy and prognostic evaluation [4]. Despite this diagnostic progress, the mechanisms driving CTCLs are still unknown.

Recent evidence highlights the pivotal role of the Tumor Microenvironment (TME) in pathogenesis and progression of CTCL suggesting a complex interplay between genetic factors and the immune system [5]. The skin areas, where tumorigenesis takes place, are infiltrated by a diverse array of immune and non-immune cells, which collectively modulate the secretory milieu [6]. Understanding the components of the TME holds promise for decoding the balance between tumor promotion and suppression, thereby guiding therapeutic strategies [5–7]. In this review, we aim to elucidate the intricate involvement of the cellular TME in CTCL appearance and progression and highlight the potential of cell profiling in optimizing therapeutic outcomes in CTCL patients.

## Key Players in the TME and Their Interactions

As listed in the Table 1 the TME of CTCL includes a profusion of immune and non-immune cells.

**Table 1:** The different cell types and their main functions in the TME of CTCL

Cell Type	Main Functions in TME of CTCL
T-Cells	- **CD4 + T helper cells**: dysregulation of Th1/Th2 balance as the disease progresses, making the immunosuppressive Th2 environment prevalent and thus the immune evasion of tumor
	- **CD8 + cytotoxic T-cells**: exhausted, especially in late stages, contributing to tumor progression
	- **T regulatory cells** (**Tregs)**: elevated numbers of unconventional Tregs possibly due to the immunosuppressive environment which they promote
B-cells	- **B cells**: elevated number of CD20 + B cells as the stages of MF develop and excessive production of IgE and IgG4 as a response to Th2 cytokines
	-**B regulatory cells (Bregs)**: low levels of IL-10 B regulatory cells in advanced MF and the subsets which promote them
Macrophages	- **M1**: antigen presentation, anti-tumor effects in the beginning of CTCL, activating Th1 response
	- **M2**: tumor-promoting functions including immunosuppression and angiogenesis, activating Th2 response
Fibroblasts	- induce immunosuppression through the STAT3-CCL2 signaling pathway - shield early-stage MF malignant cells from the anticancer drug doxorubicin
Dendritic Cells	- elevated plasmacytoid DCs, epidermal LCs and three subsets of dermal DDCs - mainly immature as the disease progresses, incapable of antigen presentation to Th1 cells but activating Tregs, inducing tolerance
Keratinocytes	- excessive proliferation due to IL-25 and TSLP secretion - promote Th2 cytokine production - hyperplasia, driven by STAT3 upregulation because of malignant T-cell cytokines’ release
Endothelial Cells	- promotion of angiogenesis, facilitating tumor growth through the upregulation of VEGFs-VEGFRs and massive expression of IL-6 and LTα
Myeloid-derived Suppressor Cells (MDSCs)	- elevated G-MDSCs in late stages MF inducing immune tolerance of tumor cells - proposed as biomarkers for CTCL progression
Natural Killer Cells	- elevated numbers in CTCL blood samples associated with poor prognosis - interactions between NK and CTCL cells contribute to aberrant function
Eosinophils	- scarce in cases of patch and plaque MF - abundant in tumorous lesions and alongside with IgE levels, serve as a prognostic factor
Neutrophils	- activated in the blood in early stages possibly due to the high production of IL-8 by T cells - few inactivated numbers of them in lesions of SS patients due to aberrant IL-17 expression
Mast Cells	- increased in the periphery of CTCL tumors - strongly correlated with disease progression - tumor growth promotion though the release of proangiogenic factors (VEGFs)

### T-Cells

#### CD4 + T-Cells

##### Th1/Th2 (T Helper) Cells

In the early stages of MF, there are benign Th1 type infiltrates secreting IL-2, IFN-γ, IL-12 and TNF-β and recruiting macrophages. This pro-inflammatory biased cytokine pattern represents a robust anti-neoplastic immune response against the malignant population [8]. On the other hand, in the advanced-staged MF and SS defects in the production of Th1 cytokines lead to a Th2 dominated TME with cytokines such as IL-4, IL-5, IL-10 and IL-13 that promote heavy humoral immunity with IgE and IgG4 isotype switching [9]. This alteration in T helper cells may have a significant impact on therapeutic choices since treatments with IL-12 [10] and interferons α and γ (IFN-α, IFN-γ) [11] have shown improvement in skin lesions by favoring Th1 response suggesting that these cytokines combat the transition to Th2 in favor of Th1 [10, 11]. At the same time, an opposite effect occurs -acceleration of the symptoms- after administration of an agent blocking IL-4 (dupilumab) posing a challenge to the Th1/Th2 imbalance hypothesis [12]. Thus, it is not clear how this fits with the hypothesis that MF and SS is a Th1/ Th2 imbalance.

##### Regulatory T-Cells (Tregs)

According to Krejsgaard et al.. in patch and plaque-staged MF the proportion of Tregs in the benign lymphocytic infiltrates remain constant. In the tumor stage, however, there is an abrupt decrease of Tregs with a concomitant increase in the number of malignant T cell supporting the antitumor role of Tregs [13]. While, in 2017 Geskin et al. proposed the existence of both conventional and unconventional Tregs highlighting the possibility of malignant CD4 + cells to adopt a Treg phenotype and that measuring Tregs as malignant cells is not uncommon [14]. Nevertheless, further elucidation is required to fully understand the role of Tregs in MF/SS progression.

#### CD8 + T-Cells

In CTCL, the CD8 + T-cells or Cytotoxic T Lymphocytes (CTLs) behave destructively, diminishing within the lesions as the disease progresses, influenced by Th2 cytokines [15]. In the early stages of MF CD8 + populations are partially activated, but this activation may not be geared towards tumor cell elimination but rather towards adopting an exhaustion phenotype, characterized by heightened expression of the triad PD-1, TIGIT and TIM-3 [16]. This exhaustion phenotype regulates the expression of thymocyte-selection-associated high-motility group box protein (TOX), which levels are elevated in CTCL cells based on gene profiling and this is strongly associated with poor prognosis [17]. Klemke et al. in 2015 proposed that PD-1 can serve as a distinguishing factor between SS and other inflammatory dermatoses as the former possess higher expression of PD-1 on CD4 + and CD8 + T cells [18]. Although, CD8 + T-cell infiltration of TME correlates with a better prognosis [15], further investigation is warranted, as there may be variations among patients [19].

### Macrophages

M1 macrophages, recognized for their pro-inflammatory role, secrete high levels of IL-12 and IL-23, which activate Th1 responses and exert anti-tumor effects. On the other hand, M2 polarization results in the formation of tumor-associated macrophages (TAMs) driven by the predominance of IL-4, IL-10, and IL-13 production from activated Th2 cells [20].

In the TME of MF and SS lesions, the abundance of M2 macrophages plays a critical role in tumor development. Inhibition or depletion of these macrophages leads to better prognosis [21, 22]. Skin biopsies from CTCL patients reveal a significant increase in the number of M2 macrophages expressing markers such as CD163 [23], CD63 [24], or CD68 [25].

### B-Cells

In 2021 Nielsen et al., performing NanoString for gene expression analysis found that the *MS4A1* (Membrane Spanning 4-Domains A1) gene was upregulated in advanced MF patients’ biopsies. In humans CD20 is encoded by the *MS4A1* gene so they went further, and their immunofluorescence analysis showed that PAX5^+^ CD20^+^ B cells were elevated [26], which contrasted with the study of 2016 conducted by Iliadis et al. [27]

#### Regulatory B-Cells (Bregs)

In CTCL, the role of Bregs remains poorly understood. Evidence suggests that Bregs may be implicated in disease progression, as advanced stages of MF show reduced IL-10 producing Bregs [28].

### Fibroblasts

Fibroblasts promote tumor growth and metastasis through the secretion of various cytokines and therefore named cancer-associated fibroblasts (CAFs) [29]. Fibroblast activation protein-α (FAP-α), a serine protease, is notably overexpressed in fibroblasts of MF lesions. It is implicated in inducing immunosuppression through the STAT3-CCL2 signaling pathway [30]. Additionally, a study in 2021 suggested that CAFs shield early staged MF malignant cells from the anticancer activity of doxorubicin by upregulating the ligand CXCL12 on CAFs, which binds to the CXCR4 receptor on cancer cells [31].

### Dendritic Cells (DCs)

DCs exhibit heterogeneity, with four distinct categories identified: conventional DCs (comprising of cDC1 and cDC2 subsets), plasmacytoid DCs (pDCs), inflammatory DCs, and resident skin DCs including epidermal (LCs) and dermal DCs [32]. Schwingshackl et al. in 2012 revealed an increase in the numbers of pDCs, LCs, and DDCs, with most of them being phenotypically immature, suggesting an interaction between malignant cells and DCs that inhibits their differentiation [33]. This finding further supports the hypothesis put by Schlapbach et al. two years earlier, that there is a link between DC maturity and tumor progression [34]. It is possible that the tumor-derived interleukin IL-10 to facilitate the aberrant DC maturation [35].

### Keratinocytes

Keratinocytes activate pro-inflammatory cytokine secretion and recruit Th1 cells, aiding in anti-tumor activity [36]. Skin biopsies from MF and SS cutaneous lesions suggest potential interactions between keratinocytes, fibroblasts, and both malignant and non-malignant T-cells, contributing to an unfortunate immunosuppressive environment [37].

Additionally, in CTCL lesions, Th2 cells produce IL-4 and IL-13, leading to upregulation of the POSTN gene in fibroblasts, which encodes periostin. This, in turn, synergistically enhances IL-25 and thymic stromal lymphopoietin (TSLP) secretion from keratinocytes, upregulating the STAT5 gene in malignant T-cells [38–40]. Consequently, an excessive proliferation of the latter and Th2 cytokine production is promoting [39]. Keratinocyte hyperplasia, driven by STAT3 upregulation, or compromised skin-barrier function due to decreased production of proteins like filaggrin, loricrin, and antimicrobial peptides (AMPs) at the mRNA expression level, reflect the impact of malignant T-cell production of IL-4, IL-13, IL-22, and their subsequent effects on keratinocytes [41, 42].

### Endothelial Cells (ECs)

Angiogenesis, crucial for tumor growth, is heightened in CTCL [44], as it is supported by elevated levels of CXCL12-CXCR4 [45] and Ang2-Tie (Angiopoietin 2-tyrosine kinase receptor) [46] signaling during MF and SS, respectively, facilitating EC activation. This results in an increased EC count [44–46].

Vesicular Endothelial Growth Factors (VEGFs) A, B, C, and D when binding to their respective receptors (VEGFRs) aid EC proliferation. These factors are significantly upregulated in late-staged CTCL skin lesions and particularly VEGFC which is responsible for lymphangiogenesis and subsequent lymph node metastasis [47, 48].

In situ studies of malignant T-cells in CTCL demonstrate strong expression of Lymphotoxin α (LTα) due to aberrant activation of the JAK3/STAT5 pathway, stimulating autocrine secretion of IL-6. Thus, ligands and receptors involved in angiogenesis and lymphangiogenesis, along with IL-6 and LTα, collaboratively promote new vessel formation, presenting potential therapeutic targets for future research [48].

### Myeloid-Derived Suppressor Cells (MDSCs)

MDSCs are distinguished by their suppressive potential, earning them a characterization as pro-tumorigenic [49]. Despite their importance, little is known about MDSCs in CTCL. A 2020 study proposed a novel and easily assessable biomarker for MF, correlating the disease stage with the increase of MDSCs and particularly the Granulocytic subset (G-MDSCs), one of two MDSC categories resembling neutrophils with a CD11b + CD14- CD15 + phenotype [50].

### Natural Killer Cells (NKs)

The importance of NKs as effector cells in tumors cannot be overstated [50–52]. This is attributed to the high levels of IL-15 from CD4 + T-cells in skin lesions [51], leading to phosphorylation of STAT5 (pSTAT5) in NK cells [52]. Consequently, elevated numbers of NK cells in CTCL blood samples are associated with poor prognosis [51, 52]. Scheffschick et al. in 2023 demonstrated that interactions between NK and CTCL cells contribute to aberrant function, despite their inherent capacity. The impaired elimination of diseased cells is attributed to the distinct cellular and molecular identity of the skin, which hinders NK cell effectiveness [53].

### Eosinophils

Eosinophils respond to stimulation by IL-4, IL-9, and IL-13 secreted by Th2 cells [54]. Interestingly, they are notably scarce in cases of patch and plaque MF [55], but elevated levels of IgE and eosinophils may serve as prognostic factors, indicating disease progression [56, 57]. In CTCL skin, eosinophils respond to IL-5 and High-mobility group BOX-1 protein (HMGB1) derived from T-cells, leading to their accumulation in lesions. The combined activation of STAT3 and STAT5 is responsible for the attraction of the eosinophils to the lesions and could potentially be targeted therapeutically [58]. Further investigation into the role of eosinophils in promoting tumor growth is warranted, as their presence is beneficial for both diagnosis [59] and prognosis [57].

### Neutrophils

Neutrophils, constituting the largest percentage of leukocytes in the blood, serve as the frontline defenders of the immune system [60]. In CTCL, even in the early stages, there is a notable activation of neutrophils in the blood [61]. This, coupled with elevated levels of the T-cell secreted mediators IL-8 and Leukotriene B4 (LTB4), which both activate and guide neutrophils, suggests a possible T cell-neutrophil communication involved in the CTCL pathology [61, 62].

Moreover, evidence suggests that aberrant expression of IL-17 from malignant T-cells in MF [63] and SS [64] lesions, rather than from conventional Th17 cells, recruits neutrophils [63, 64]. However, these neutrophils appear to be functionally impaired, characterised by abnormal stimulation and they cannot effectively defend the host against pathogens [64].

Currently, efforts are underway to prognose MF using the Neutrophil-to-Lymphocyte Ratio (NLR) [65].

### Mast Cells

Mast cells appear significantly increased in the periphery of CTCL tumors and strongly correlate with disease progression [66]. Additionally, they promote tumor growth by releasing pro-angiogenic factors like VEGFs packaged in their secretory granules [67].

## Therapeutic Approaches Regarding the TME

Providing therapy to CTCL patients undoubtedly is a challenge for physicians. As CTCL is a heterogeneous and versatile clinical entity with individual needs for each patient, the personalized therapeutic approach is necessary [68]. However, recent evidence and continuously conducted clinical trials suggest that novel therapies targeting different components of the TME are promisingly effective as shown in Table 2 [5–7].

**Table 2:** The different therapies and their mechanism of action depending on the TME

Category	Drug	Mechanism / Target
Monoclonal Antibodies (mAb)	Alemtuzumab	Targets CD52, induces lymphopenia via complement activation and ADCC
	Brentuximab Vedotin	Binds to CD30, delivers MMAE to induce apoptosis
	Mogamulizumab	Binds to CCR4, induces ADCC
	Lacutamab (IPH4102)	Targets KIR3DL2, induces malignant cytolysis through ADCC and Fcγ R-mediated phagocytosis
	Zanolimumab	Binds to CD4, prevents interaction with MHC-II, activates ADCC
Immune Checkpoint Inhibitors (ICIs)	Pedrolizumab	Targets PD-1/PD-L1 to restore immune responses
	Nivolumab	Targets PD-1/PD-L1 to restore immune responses
	Atezolizumab	Targets PD-L1 to restore immune responses
	Durvalumab	Targets PD-L1 to restore immune responses
	Ipilimumab	Blocks CTLA-4 to enhance T cell activation
	TTI-621	Blocks CD47-SIRPα axis to enhance phagocytosis
Immunotoxins (ITs)	Denileukin diftitox	Targets CD25, induces apoptosis after internalization
	CCR4-IL2 IT	Targets CCR4 and CD25, induces apoptosis after internalization
	Resimmune	Targets CD3ε, induces apoptosis of neoplastic T-cells
Toll-Like Receptor (TLR) Agonists	Imiquimod	Agonist of TLR-7, activation of innate (direct) and adaptive (indirect) immunity
	Resiquimod	Agonist of TLR-7/8, activation of innate (direct) and adaptive (indirect) immunity
	CpG ODN	Agonist of TLR-9, activation of innate (direct) and adaptive (indirect) immunity
mTOR Inhibitors	Everolimus	Inhibits mTORC1, induces apoptosis of cancer cells
Mucin 1 (MUC1) Inhibitors	GO-203	Targets MUC1-C, inhibits metastasis and apoptosis inhibition
Histone Deacetylase (HDAC) Inhibitors	Vorinostat	Inhibits class I and II HDAC, resulting in cell cycle arrest and apoptosis
	Romidepsin	Inhibits class I HDAC, resulting in cell cycle arrest and apoptosis
	Resminostat	Inhibits class 1, 2b and 4 HDACs, resulting in cell cycle arrest and apoptosis
Proteasome Inhibitors (PIs)	Bortezomib	Inhibits chymotrypsin and caspase of proteasome, NFκΒ activation and induces apoptosis of cancer cells
B cell therapy	Rituximab	Targets CD20, activates complement and ADCC inducing apoptosis of early B cells
Others	Dimethyl Fumarate (DMF)	Suppresses NF-κB, induces apoptosis of CTCL cells
	Lenalidomide	Increases CD8, CD25, and FoxP3 expression with decreased CD4:CD8 ratio
	JAK-STAT inhibitors	Inhibit JAK or STAT proteins in the JAK-STAT pathway, immunomodulatory effects
	Mast cell inhibition	Elimination, modulation of phenotype or altering the actions of secreted products of malignant cells
	CAR T cell therapy	Utilizes chimeric antigen receptors to target specific antigens and eliminate cancer cells

### Mononoclonal Antibodies (mAbs)

Monoclonal antibodies are immunoglobulins (Igs) with monovalent affinity, binding only to the same epitope and produced by a specific clone of plasma cells [69].

#### Alemtuzumab

CD52, a GPI-anchored glycoprotein, is a widely expressed cell surface Ag found at high levels in normal and malignant peripheral T (CD3+) and B (CD19+) cells and at lower levels in NKs, monocytes, and macrophages [70]. Alemtuzumab is a humanized mAb binding to the human CD52 protein. After infusion it causes depletion of CD52-expressing T-cells, B-cells, NKs leading to a profound lymphopenia [71]. A 10-year study revealed its effectiveness in inducing a long-term remission in SS but not in MF patients. This differentiation may be due to the different origins of malignant T cells in the two clinical entities [72]. Α Phase II clinical trial for Alemtuzumab in CTCL patients has been completed without published results so far [73, 74].

#### Brentuximab Vedotin

CD30 is a transmembrane protein of the TNF superfamily which is highly expressed on lymphoma cells. CD30 is the target of brentuximab vedotin, a conjugated Ab composed of a chimeric IgG1 covalently linked to monomethyl auristatin E (MMAE). Following binding to CD30 and its endocytosis, MMAE is released in the cytosol disrupting the microtubule network and causing apoptosis [74]. Since 2017, it is FDA approved for CD30 + MF patients who have already received prior systemic treatment [75].

#### Mogamulizumab

The two CC chemokines CCL17 and CCL22 bind to their receptor CCR4 which is mainly expressed by Th2, Th17 and Treg cells, regulating their migration to the skin [76]. CCR4 is highly expressed on malignant T-cells in MF cutaneous lesions and on circulating malignant T-cells making it an ideal target for treatment [77]. A non-fucosylated, humanized IgG1κ named mogamulizumab, had been approved in 2018 for relapsed/refractory MF or SS. It selectively binds to the N-terminus of CCR4 and induces strong ADCC against CCR4 + malignant T-cells [78].

#### Lacutamab (IPH4102)

Killer-cell immunoglobulin-like receptors (KIRs) are cell surface receptors of NKs which bind to the MHC-I molecules of virally infected or transformed cells in order to eliminate them. Most of the KIRs are inhibitory receptors, hindering the cytotoxic effects [79]. A member of these inhibitory receptors, KIR3DL2, also known as CD158k, is highly expressed in MF and SS cells, emerging it as a diagnostic marker [80]. Therefore, lacutamab, a humanized IgG1 mAb targeting KIR3DL2 is currently under a multi-cohort, phase 2 trial [81].

#### Zanolimumab

CD4, the cell surface glycoprotein of Th cells is important for the enhancement of the TCR signals as its α_2_ subunit interacts with the MHC-II molecules [82]. This interaction is prevented by Zanolinumab, a human IgG1κ which binds to CD4, leading to inactivation and even apoptosis of CD4 + T-cells -particularly the CD45RA + ones- which are present in most of the cases of CTCL. There is a completed phase 2 clinical trial about Zanolimumab in CTCL patients [83].

### Immune Checkpoint Inhibitors

Immune checkpoints are membrane receptors that turn on or off the immune responses, depending on the environment and the cellular circumstances. Immune Checkpoint Inhibitors (ICIs) are drugs mainly comprised of mAbs that target these receptors in favor or against the immune responses, depending on the disease [84].

#### PD-1/PDL-1 (Programmed Cell Death-1/Programmed Cell Death Ligand-1)

PD-1 is a receptor expressed on activated T and B cells, NKs, DCs, macrophages, monocytes and extremely on malignant T cells. PD-L1 (also known as B7-H1) is expressed on the surface of macrophages, some activated T and B cells, DCs and some epithelial cells. Apart from the immune evasion of malignant cells that occurs after the binding between the PD-1/PD-L1 as a result of the T-cell anergy, their interaction acts also protumorigenic with proliferative and survival signaling effects in the cancer cells [85].

Studies have been conducted for the understanding of PD1/PD-L1 axis in cutaneous lymphomas resulting in contradictory conclusions. It is supported that PD1 but not PD-L1 is highly expressed in malignant T cells of CTCL lesions. Furthermore, in MF malignant cells PD-1 is expressed at a lower proportion compared to SS supporting the consideration of them as two distinct entities and possibly correlating with the immunosuppression that gradually occurs in advanced stages [86]. Therefore, the axis PD-1/PD-L1 can serve as a prognostic factor [87]. In 2020, Saulite et al., showed that the PD-1 blockade in ex vivo studies of SS patients reversed the impairment of the immune system by producing IFN-γ and boosting the proliferation of both Th1 and tumor cells [88]. This is consistent with the hyperprogression of MF in a patient after anti–PD-1 treatment [89].

#### CTLA-4 (Cytotoxic T-Lymphocyte Associated Protein 4)

CTLA-4 is a coinhibitory receptor and its engagement to its ligand B7 hinders the activation of T cells. Chemical induction of CTLA-4 expression on PBMCs from MF patients was more profound compared to normal ones [90]. In 2019, Anzengruber et al., showed that the SS malignant T-cells did not demonstrate extreme differences in the expression levels of CTLA-4 between the tumor, non-tumor cells, and healthy controls [91] which was in contrast with previous studies displaying that CTLA-4 was upregulated in MF and SS patients and correlates with disease progression [90, 92]. To date, there are two case reports treated with Ipilimumab, a fully human mAb IgG1κ against CTLA-4. The first one was a male MF patient in his early forties with fully remission of MF cutaneous lesions during treatment with ipilimumab for advanced melanoma [93] and the second one was the case of a 67-year-old female SS patient who highly expressed CTLA4-CD28 fusion in the cancer cells, a finding that permitting treatment with ipilimumab [94].

#### CD47

Signal regulatory protein α (SIRPα) and CD47 engagement develops the “don’t eat me” signal to the macrophages preventing phagocytosis. This results in tumor immune escape and cancer progression. Therefore, mAbs and antagonists for the CD47-SIRPα axis are investigated in both pre-clinical and clinical level for CTCL.

In 2023, Han et al. used TTI-621, a recombinant fused protein, in human CTCL cells. This treatment blocks the “don’t eat me signal” through the human SIRPα N-terminal domain and delivers a strong “eat me signal” through the Fc receptor of IgG1. As expected, they found improved phagocytosis of the tumor cells. It can also be combined with PD-L1 inhibitors for better therapeutic outcomes [95].

A phase I clinical trial of TTI-621 where CTCL patients received intratumoral injections of the drug revealed that both numbers of circulating Sézary cells and tumor size were decreased through the activation of the anti-tumor immunity [96].

Thrombospondin-1 (TSP) is another ligand of CD47 with serum levels less increased in MF patients compared to SS ones. However, it is a proper target for future investigation as TSP is responsible for tumor immunotolerance in various types of cancer [97].

### Immunotoxins (ITs)

Immunotoxins are anticancer agents composed of a mAb fragment that is fused to a protein with cytotoxic effects. After binding to a specific receptor and their internalization in the target cells, they induce apoptosis [98].

#### Denileukin Diftitox

After pivotal clinical trials, FDA approved denileukin diftitox as a treatment for patients with relapsed/refractory CTCL following one or more prior lines of systemic therapy. This drug is a fused protein of IL-2 with diphtheria toxin and targets the CD25 subunit of IL-2 receptor (IL-2R) of the target cells. Upon the internalization via receptor-mediated endocytosis, delineukin diftitox causes the apoptosis of the malignant cells and reduced MDSC function, suggesting a potential mechanism of action of this drug and emerging the importance of targeting MDSCs [99].

#### CCR4-IL2 IT

CCR4-IL2-diphtheria toxin (CCR4‐IL2 IT) targets both CCR4 and CD25. With this mechanism of action, it causes depletion of CCR4 + and CD25 + CTCL cells and tumor‐infiltrating effector Tregs boosting the patient’s anticancer immune response. According to a study by Wang et al. in 2023, CCR4‐IL2 IT demonstrated higher rates of prolonging survival than brentuximab vedotin in a CTCL mouse model [100].

#### Resimmune

Resimmune is derived from diptheria toxin against the extracellular domain of CD3ε chain, a costimulatory molecule of T-cells. In 2015, Frankel et al. showed that CTCL patients of intermediate stage highly responded to Resimmume. However, there are no recent data on its efficacy and safety profile, putting the necessity for further clinical trials [101].

### Toll-Like Receptor (TLR) Agonists

Toll-like receptors are members of the Pattern Recognition Receptors (PRRs) and belong to the innate immune system, recognizing danger molecules. Activation of the intracellular or cell membrane TLRs leads to expression of pro-inflammatory and type-I IFN (IFN-α/β) genes stimulating both innate and adaptive immunity. Therefore, TLR agonists are promising agents in the CTCL therapy as they can activate cytotoxic cells [102].

TLR7 could serve as a prognostic factor in MF due to the lower expression in MF skin samples [103]. **Imiquimod**, an immune response modifier, is a TLR-7 agonist. Topical imiquimod is effective in localized lesions of a significant portion of early-staged MF patients by stimulating the secretion of IFN-α, TNF-α, IL-1, and IL-6, cytokines with anti-tumor activity [104].

A 2015 clinical trial about **Resiquimod**, an agonist of TLR-7/8 which stimulates the production of IFN-α, IL-12, and IL-15 with possible systemic activity even if applied topically, showed significant shrinkage of skin lesions in early CTCL and better T-cell anti-tumor activities [105].

**TLR-9 agonist**, a synthetic oligonucleotide with cytidine-phospho-guanosine (CpG) motifs (CpG ODN), demonstrated induction of CD8 + T cells and systemic tumor regression in MF patients [105].

### mTOR Inhibitors

The mammalian target of rapamycin (mTOR) is a protein kinase that plays a pivotal role in key functions implicating in cancer pathogenesis and therefore comprises a therapeutic target. A phase 2 trial, where MF patients were administered oral **Everolimus**, an mTORC1 inhibitor, showed response rates of 43% with good safety profile [106]. However, further clinical trials are necessary.

### Mucin 1 (MUC1) Inhibitors

Mucin 1 in normal cells, it is a cell surface barrier acting as a moisturizer while in cancer cells is overexpressed and responsible for the metastasis and inhibition of apoptosis [107]. According to Jain et al., treating CTCL with MUC-1 inhibitor leads to apoptosis of cancer cells, emerging it as a potential therapeutic target [108]. The same group in 2017 demonstrated that combination of Decitabine, an hypomethylating agent with GO-203, a MUC1 inhibitor, heightened the ROS levels and led to CTCL cell death [109].

### Histone Deacetylase (HDAC) Inhibitors (HDIs)

Histone deacetylases are enzymes which remove acetyl groups from histones remodeling chromatin, making it not accessible to transcription factors and thus gene expression is hindered. Genomic analysis of tumor cells revealed that HDACs operate in favor of cancer and targeting them is a good therapeutic option [110].

#### Vorinostat

Vorinostat, also known as suberoylanilide hydroxamic acid (SAHA), is class I and II HDI that leads to the accumulation of acetylated proteins and thus in the cell cycle arrest, apoptosis and inhibition of angiogenesis. Since 2006 it has been approved by FDA for patients with refractory CTCL who have received at least two prior systemic therapies including bexarotene. The primary toxicities include nausea, vomiting, diarrhea, fatigue, and a transient decrease in hemoglobin, platelet and white cell counts [111].

#### Romidepsin

In 2009 FDA approved Romidepsin, a cycodepsipeptide obtained from the bacteria *Chromobacterium violaceum*, as a class I selective HDI for the treatment of adult patients with CTCL who have received at least one prior systemic therapy. Romidepsin results in cell cycle arrest and apoptosis downregulating the expression of pro-survival genes [112].

#### Resminostat

Currently, a double blind, placebo-controlled phase II trial, the RESMAIN study, has been completed evaluating the Resminostat, an orally hydroxamic acid class 1, 2b and 4 HDI for maintenance treatment of patients with advanced stages of MF or SS that have achieved disease control with systemic therapy [113].

### Proteasome Inhibitors (PIs)

Proteasome is a complex cellular machine comprised of catalytic (proteases) and regulatory subunits. It is responsible for the turnover of unnecessary proteins and thus the cell survival. Therefore, inhibition of proteasome leads to a toxic protein burden and consequently in the cell death of cancer cells [114].

#### Bortezomib

Bortezomib is the first FDA-approved PI that inhibits both proteasome and the activation of NF-κΒ leading to apoptosis of neoplastic cells [114]. Since the pathway of NF-κB is aberrant in CTCL, this agent could serve as a promising medicine in this lymphoma category [115].

Several studies have evaluated the efficacy of Bortezomib in CTCL. A 2015 in vitro study showed that Bortezomib inhibits the expression of TGF-β1, IL-10 and CXCR4, key components of the Th2 biased response decreasing the CTCL cell survival [116]. Further studies proposed that Bortezomib combined with methotrexate or SAHA induces apoptosis of CTCL cells [117].

Moreover, a phase 2 clinical trial of Bortezomib as monotherapy in CTCL showed response rates of approximately 70% with a good safety profile with mild adverse effects [118]. The same toxicities were noticed when it was combined with Romidepsin [119].

### B Cell Therapy

CD20 while it is generally a B-cell marker B there are published cases of MF patients with some CD20 positivity in the T-cell infiltration of lymphoma lesions. Some of them were treated with Rituximab, a humanized chimeric anti-CD20 mAb, without achieving remission of the disease despite the depletion of surface antigen CD20 on the histopathology Sect. [120]. Further studies will shed light on the use of B cell depletion therapies in CTCL.

### Other Therapies

#### Dimethyl Fumarate (DMF)

Immunomodulation is probably the main activity of Dimethyl fumarate which induces apoptosis of CTCL cell lines via suppression of NF-κΒ. A multicenter phase 2 trial found approximately the same response rates in both MF and SS patients but higher efficacy in the late-stage CTCL skin. These findings alongside with its excellent tolerability make DMF a promising therapy for CTCL and clinical trials must be carried out combining it with other drugs for even better outcomes [121].

#### Lenalidomide

An open-label multicenter phase 2 trial of lenalidomide, a derivative of thalidomide, demonstrated its efficacy in approximately one-third of CTCL patients who achieved partial response. Lenalidomide is thought to induce changes in cytokine and cellular TME as it appears to decrease circulating CD25 + and CD4 + T cells in the peripheral blood. In the skin, lenalidomide induces an increase of CD8, CD25 and FoxP3 expression with decreased CD4:CD8 ratio [122]. However, the exact mechanism in the CTCL remains unclear.

#### Janus Kinase-Signal Transducer and Activator of Transcription Inhibitors (JAK-STAT Inhibitors)

The JAK-STAT signaling pathway is dysregulated in CTCL patients [43, 64] making it a promising target. A 2024 systematic review highlighted the need for larger prospective randomized clinical trials of JAK/STAT inhibitors in CTCL as the response rates in MF reached even 45% with acceptable side effects [123]. Even though, in vitro and in vivo studies of STAT inhibitors have been conducted for many types of cancer, without any of them being cutaneous lymphoma [124].

#### Mast Cell Inhibition

Therapies regarding mast cells examine elimination, promotion of their anti-tumor activity or alteration of the effect of their secreted products [125]. Currently, there are no available pre- or clinical data for the previously mentioned approaches in CTCL.

#### CAR (Chimeric Antigens Receptor) T Cell Therapy

CAR T cells need to be investigated in CTCL as the therapeutic outcomes for hematological malignancies are promising. CAR T cell therapy basic ides is the isolation of T cells from patients and genetically modify them ex vivo so as to express receptors that recognize specific antigens on the surface of cancer cells and after infusion back to the patients to eliminate them. A 2022 review analyzes six potential antigens targeted by CAR-T cells in CTCL, namely CD4, CD47, CD30, CCR4, TAG-72, and CD37 and examines the limitations of CAR T cell therapy [126].

## Conclusion

Undoubtedly, the emerging role of the TME in the progression of CTCL has become a focal point for research. In addition to genetic factors, the TME in Mycosis Fungoides (MF) and Sézary Syndrome (SS) is closely linked to the transition from early to late stages of the disease. The TME milieu predominantly promotes tumor growth through immunosuppressive cellular and molecular mechanisms. Consequently, treatments are aimed at shifting towards pro-inflammatory mechanisms and eliminating malignant cells.

**Fig. 1:**
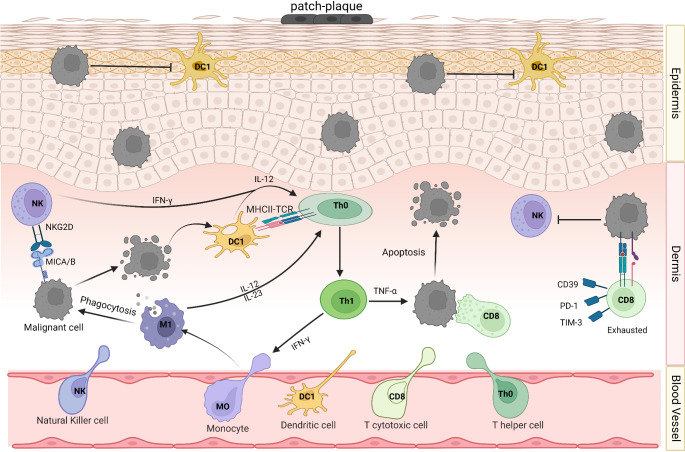
Early stages of CTCL. As the early stages of CTCL arise, a small number of malignant cells are distributed in the epidermis and dermis of patch-plaque lesions. In the epidermis, the resident Dendritic Cells type 1 (DC1) become inactive upon their encounter with malignant cells. In the dermis, the infiltration of a plethora of immune cells such as monocytes/macrophages, Natural Killer cells (NKs), Dendritic cells (DCs), CD4 + and CD8 + T cells, manage to kill some malignant cells. For example, NKs recognize the tumor cells after the engagement of NK2GD-MICA/B resulting in their apoptosis. In parallel, DCs present tumor antigens to the Th0 (T-helper 0) cells turning them to the pro-inflammatory Th1 (T-helper 1) phenotype which release TNF-α and alongside with the active CD8 + T cells further kill malignant cells. IFN-γ released by NKs and Th1 cells helps the transition M0 to M1 macrophages which phagocytose tumor cells and serve as pro-inflammatory. In addition, M1 and mature DCs not only release pro-inflammatory cytokines (IL-12, IL-23) turning Th0 to Th1 but also present tumor antigens through MHC-I and -II to CD4 + and CD8 + T cells, respectively, inducing their cytotoxic activity against tumor cells. However, sometimes the cytotoxic cells (CD8 + T-cells and NKs) interact with malignant cells in order to eliminate them, but the immunosuppressive effects of the latter make the former paralyzed. As a result, CD8 + T-cells exhibit an exhausted phenotype as indicated by upregulation and high expression of CD39, TIM-3, PD-1 inhibitory receptors on their cell surface

**Fig. 2:**
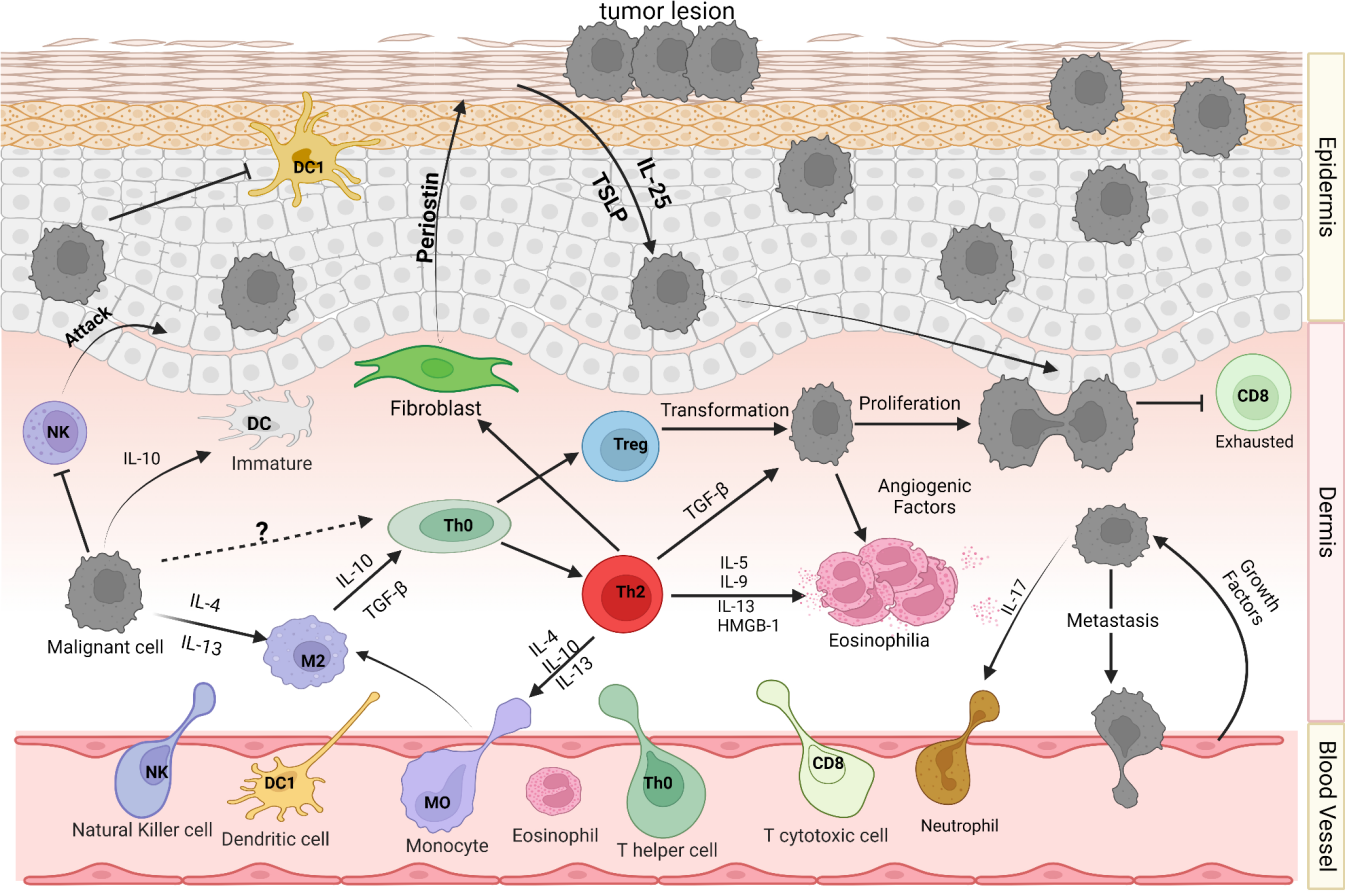
Late stages of CTCL. In the late stages of CTCL, the tumorous lesions are characterized by a highly immunosuppressive environment where both epidermis and dermis are becoming fraught with tumor cells that exhaust CD8 + T cells after their constant interaction with them. In the dermis, the DCs are mainly immature and cannot phagocytose the debris of the apoptotic cancer cells obtained from the scarce activity of the NKs which start to attack normal cells due to the distorted architecture of the skin. The malignant environment induces Th2 (T-helper 2) and T-regulatory cells (Tregs) which operate in favor of the immune evasion and proliferation of the tumor cells through the production of the anti-inflammatory cytokines IL-5, IL-9, IL-13, TGF-β. There are indications also of Treg transformation into malignant cells but remains to be elucidated further. In parallel, Th2 cells induce the production of periostin from fibroblasts which in turn upregulates the production of IL-25 and Thymic stromal lymphopoietin (TSLP) from keratinocytes, which act again in favor of cancer proliferation. Moreover, Th2 and malignant cells recruit eosinophils in the dermis after the secreting of anti-inflammatory cytokines and pro-angiogenic factors, respectively. Eosinophils’ accumulation and high activation, a phenomenon named eosinophilia, maybe is one of the pruritus’ culprits. Additionally, neutrophils’ recruitment is due to the IL-17 production of malignant cells instead of the T-cells derived one. Lastly, the growth factors secreted from the endothelial cells lead to further cancer development and even metastasis in the bloodstream

To conclude, tumor cells interact with infiltrating cells, and although these cells may initially possess anti-tumoral capabilities, they become paralyzed after manipulation by malignant cells. Understanding the complexity of these interactions both in early (Fig. 1) and late stages of CTCL (Fig. 2) and the precise phenotype of immune cells within lesions enhances our knowledge of potential targets for future treatments and personalized therapies tailored to the TME of each patient. Moreover, the challenging differentiation of MF/SS from other skin diseases poses a dilemma for physicians, even after various histopathological and molecular assays have been conducted. Therefore, precise molecular identification of malignant cell populations and other cell types within the TME lays the groundwork for more accurate diagnosis, prognosis, and treatment of the disease, considering the heterogeneity of CTCL.

## Key References

- Boutilier AJ, Elsawa SF. Macrophage Polarization States in the Tumor Microenvironment. Int J Mol Sci. 2021;22(13):6995. doi: 10.3390/ijms22136995.
  ○ This review examines the current insights into macrophage polarization driven by the tumor microenvironment (TME) and highlights the contribution of M2-polarized macrophages to tumor progression.
- Huang S, Liao M, Chen S, Zhang P, et al. Immune signatures of CD4 and CD68 predicts disease progression in cutaneous T cell lymphoma. Am J Transl Res. 2022;14(5):3037–3051.
  ○ This is the first study that develops a tool to assess the risk of cutaneous T cell lymphoma progression by analyzing features of the skin immune microenvironment.
- Gluud M, Pallesen EMH, Buus TB, et al. Malignant T cells induce skin barrier defects through cytokine-mediated JAK/STAT signaling in cutaneous T-cell lymphoma. Blood. 2023;141(2):180–193. doi: 10.1182/blood.2022016690.
  ○ This study provides a novel rationale for the use of JAK1 inhibition as a key adjuvant therapy for individuals with cutaneous T-cell lymphoma.
- Scheffschick A, Nenonen J, Xiang M, et al. Skin infiltrating NK cells in cutaneous T-cell lymphoma are increased in number and display phenotypic alterations partially driven by the tumor. Front Immunol. 2023;14:1168684. doi: 10.3389/fimmu.2023.1168684.
  ○ This is the biggest study identifying the NK cells in cutaneous T cell lymphoma skin and investigates their phenotype and function.
- Di Raimondo C, Lombardo P, Tesei C, et al. Role of Neutrophil-to-Lymphocyte Ratio (NLR) in Patients with Mycosis Fungoides. Diagnostics (Basel). 2023;13(11):1979. doi: 10.3390/diagnostics13111979.
  ○ This is the first study assessing the neutrophil/lymphocyte ratio (NLR) with disease stage and severity in patients with mycosis fungoides.
- Han Z, Wu X, Qin H, et al. Blockade of the Immune Checkpoint CD47 by TTI-621 Potentiates the Response to Anti-PD-L1 in Cutaneous T-Cell Lymphoma. J Invest Dermatol. 2023;143(8):1569–1578.e5. doi: 10.1016/j.jid.2023.02.017.
  ○ This is the first study investigating the effects of CD47 and PD-L1 blockade in cutaneous T cell lymphoma.
- Vahabi SM, Bahramian S, Esmaeili F, et al. JAK Inhibitors in Cutaneous T-Cell Lymphoma: Friend or Foe? A Systematic Review of the Published Literature. Cancers (Basel). 2024;16(5):861. doi: 10.3390/cancers16050861.
  ○ This systematic review brings interesting results on the role of the Janus Kinase (JAK) inhibitors in the treatment of cutaneous T cell lymphoma.
- To V, Evtimov VJ, Jenkin G, et al. CAR-T cell development for Cutaneous T cell Lymphoma: current limitations and potential treatment strategies. Front Immunol. 2022;13:968395. doi: 10.3389/fimmu.2022.968395.
  ○ This is an interesting review article on CAT T cell therapy in cutaneous T cell lymphoma.

## Data Availability

This is a review article, and no new datasets were generated or analyzed during this study. All data supporting this review are cited in the manuscript and are publicly available through the respective original publications.
